# Iatrogenic Errors during Root Canal Instrumentation Performed by Dental Students 

**DOI:** 10.22037/iej.v13i1.18507

**Published:** 2018

**Authors:** Seyedeh Sareh Hendi, Hamed Karkehabadi, Amir Eskandarloo

**Affiliations:** a *Department of Endodontics, Dental School, Hamadan University of Medical Science, Hamadan, Iran; *; b * Department of Radiology, Dental School, Hamadan University of Medical Science, Hamadan, Iran*

**Keywords:** Dental Students, Procedural Errors, Root Canal Therapy

## Abstract

**Introduction:**

The present study was set to investigate the training quality and its association with the quality of root canal therapy performed by fifth year dentistry students.

**Methods and Materials:**

A total number of 432 records of endodontic treatment performed by fifth year dentistry students were qualified to be further investigated. Radiographs were assessed by two independent endodontists. Apical transportation, apical perforation, gouging, ledge formation, and the quality of temporary restoration were error types investigated in the present study.

**Results:**

the prevalence of apical transportation, ledge formation, and apical perforation errors were significantly higher in molars in comparison with other types of teeth. The most prevalent type of error was the apical transportation, which was significantly higher in mandibular teeth. There was no significant differences among teeth in terms of other types of errors.

**Conclusion:**

The quality of training provided for dentistry students should be improved and endodontic curriculum should be modified.

## Introduction

The aim of root canal therapy is to prevent and treatment of pulp and periapical diseases and to provide a condition for roots to be restored [1, 2]. The outcome of such treatments depends on the competence of clinician in performing the treatment without any error. The errors occurred during any step of treatment, including diagnosis, access cavity preparation, cleaning, shaping and root canal obturation, can jeopardize the success of the treatment [3]. For instance, the occurrence of apical transportation would result in an improper cleaning and make it difficult to fight against periapical diseases. Similarly, the apical perforation can lead to the infection of periodontal and alveolar bone [4, 5]. The success rate of primary canal treatment of teeth free of apical periodontitis in a controlled condition is 90 to 95% [6]. However, apical periodontitis normally occurs in 24.5-65.8% of all treatments performed by general dentists [6]. The majority of endodontic therapies is carried out by general dentists [7]. However, many studies have reported that a high percentage of endodontic therapies performed by general dentists do not meet the required quality, which can be due to the poor trainings they received from the endodontic department of dentistry schools [8, 9]. 

Many factors affect the training quality, including the time allocated to the theoretical and practical education during clinical and preclinical periods, teacher to student ratio, the methods employed for educating and evaluating students, the number of students, and number of patients referred to each clinic [5, 9]. 

Considering the fact that some students are not self-confident enough to perform an endodontic treatment [10], the study of treatment quality and assessment of prevalent errors (such as apical transportation, ledge formation, and apical perforation) can be useful in modifying the education curriculum and improving the competence of general dentists [11]. Accordingly, the present study was set to investigate training quality on the various types of errors committed by the fifth-year dentistry students in of Hamadan universality of medical sciences.

## Materials and Methods

In the present cross-sectional retrospective study, all records of endodontic treatments performed by the fifth-year dentistry students of Hamadan University of medical sciences during 2014 and 2015, a total number of 470 records, were investigated. This study was approved by Ethics Committee of Hamadan University of Medical Sciences. Approval number was IR.umsha.rec.1395.231. Records containing radiographs taken before treatment, during working length determination, with master cone and after treatment were included in the study. 

Moreover, the records should have followed the standard procedure mandated by the endodontic department of the Dentistry School of Hamadan University of Medical Sciences in performing endodontic treatments, otherwise they were excluded. This procedure contains the following steps; isolation with rubber dam, determination of working length with the radiograph-based bisecting angle technique, root canal preparation by step back technique with K-files (Thomas, France), using Root Canal Preparation Cream (Dentonics Monroe, NC, USA) for lubricating the canal, washing the canal with saline and 2% chlorhexidine, the use of calcium hydroxide in multi-session treatments and filling the canal in accordance with the lateral condensation filling technique using gutta-percha (GuppaDent, Tehran, Iran) and sealer with Zinc Oxide Eugenol (Associated Dental Products Ltd, Wiltshire, UK) and providing a temporary restoration with Cavisol (Aria Dent, Tehran, Iran). Furthermore, those records related to patients with an age above 68 years or lower than 16 years and those containing low-quality radiographs (insufficient contrast and density or technical errors) with superimposition of anatomical structures were excluded. Also, in Hamedan University students were not allowed to treat teeth with curved and calcified canals. Finally, 432 records were qualified to be further investigated. The clinical supervision had a teacher to student ratio of ten (*i.e.* each ten students were supervised by a teacher). Moreover, a postgraduate endodontic student continuously observed and checked the treatments provided by the fifth-year dentistry students. 

It should be mentioned that all types of teeth, including anterior, premolar, and molar were investigated by the present study. In the case of multi-root teeth, *i.e. *molars and premolars, each canal was investigated separately. Radiographs of the qualified records were assessed by two independent endodontists using a magnifier with a magnifying power of two times and a Negatoscope (LED, ajteb, 30×35, Tehran, Iran). In the cases of contradiction between what reported by two endodontists, a third experienced radiologist was asked to review the radiograph again and report the results. We evaluated errors by comparing of initial, mater cone and final radiographs. The Kappa coefficient was used to assess the agreement between endodontists.

**Table 1:** The type, location, and frequency of teeth investigated in the present study [Number (percent)]

**Type**	**location**		**Total**
	**Maxilla**	**Mandible**	
**Anterior**	102 (35.4%)	8 (5.6%)	110 (25.5%)
**Premolar**	130 (45.1%)	71 (49.3%)	201 (46.5%)
**Molar**	56 (19.4%)	65 (45.1%)	121 (28.0%)
**Total**	288 (66.67%)	144 (33.33%)	432 (100.0%)

**Table 2:** The frequency of various types of error in various types of teeth [Number (frequency)]

**Error type**		**Tooth type**			**Total**
		**Anterior**	**Premolar**	**Molar**	
**Apical Transportation**	No	106 (96.4%)	189 (94.0%)	55 (45.5%)	350 (81.0%)
	Yes	4 (3.6%)	12 (6.0%)	66 (54.5%)	82 (19.0%)
**Apical perforation**	No	97 (88.2%)	176 (87.6%)	83 (68.6%)	356 (82.4%)
	Yes	13 (11.8%)	25 (12.4%)	38 (31.4%)	76 (17.6%)
**Ledge formation**	No	109 (99.1%)	201 (100.0%)	116 (95.9%)	426 (98.6%)
	Yes	1 (0.9%)	0 (0.0%)	5 (4.1%)	6 (1.4%)
**Gouging**	No	107 (97.3%)	196 (97.5%)	115 (95.0%)	418 (96.8%)
	Yes	3 (2.7%)	5 (2.5%)	6 (5.0%)	14 (3.2%)
**Improper temporary restoration**	No	69 (62.7%)	105 (52.2%)	80 (66.1%)	254 (58.8%)
	Yes	41 (37.3%)	96 (47.8%)	41 (33.9%)	178 (41.2%)

Apical transportation, apical perforation, gouging, ledge formation, and the quality of temporary restoration were error types investigated in the present study. The iatrogenic errors were classified as: apical transportation: The obturation material is detected outside the canal walls [12], apical perforation: The obturation material is detected outside the canal wall [12], gouging: It diagnoses when there was overextension of access cavity undermining the enamel walls as apparent by radiograph [13], ledge: Root filling is at least 1 mm shorter than the working length and deviated from the original canal shape in teeth where root canal curvature occurred [12].

Once the investigation of radiographs was completed and all associated data were gathered, the data were analyzed using SPSS software (SPSS version 20, IBM Inc., Chicago, IL, USA). The *chi* square and fisher’s exact tests were employed for assessing the variables of the study.

## Results

In the present study, a total number of 432 teeth of all types of both jaws, which satisfied the inclusion criteria of the study, were selected to be investigated in terms of their endodontic treatment quality. The information of the number and type of the teeth are presented in Table 1. The frequency of various types of error in respect to teeth types is presented in Table 2. Table 3 also represents the pair comparison of teeth types in terms of various errors. 

Similarly, according to this table, the prevalence of apical transportation in molar teeth was significantly higher than those of anterior and premolar teeth. The prevalence of apical perforation was higher in molar teeth than in others.

The occurrence of ledge formation in anterior and premolar teeth were significantly higher than that of molar teeth (Table 3). 

**Table 3:** The association between various teeth types in terms of various variables based on chi-square test

**Variable**	**Association between types of teeth**	***P*** **-value**
**Apical transportation **	Anterior- Premolar	0.273
	Anterior- molar	0.000
	Premolar-Molar	0.000
**Apical perforation**	Anterior- Premolar	0.514
	Anterior- molar	0.000
	Premolar-Molar	0.000
**Ledge formation**	Anterior- Premolar	0.354
	Anterior- molar	0.043
	Premolar-Molar	0.007
**Gouging**	Anterior- Premolar	0.538*
	Anterior- molar	0.299
	Premolar-Molar	0.192
**Improper temporary restoration**	Anterior- Premolar	0.048*
	Anterior- molar	0.344*
	Premolar-Molar	0.010

**Table 4:** The frequency of various types of error in teeth based on their location [Number (percent)]

**Error type**		**Location **		**Total**	***P*** **-value**
		**Maxilla **	**Mandible**		
**Apical transportation **	No	250 (86.8%)	100 (69.4%)	350 (81.0%)	0.00
	Yes	38 (13.2%)	44 (30.6%)	82 (19.0%)	
**Apical perforation**	No	243 (84.4%)	113 (78.5%)	356 (82.4%)	0.141
	Yes	45 (15.6%)	31 (21.5%)	76 (17.6%)	
**Gouging**	No	281 (97.6%)	137 (95.1%)	418 (96.8%)	0.247
	Yes	7 (2.4%)	7 (4.9%)	14 (3.2%)	
**Ledge formation **	No	284 (98.6%)	142 (98.6%)	426 (98.6%)	1.00
	Yes	4 (1.4%)	2 (1.4%)	6 (1.4%)	
**Improper temporary restoration**	No	172 (59.7%)	82 (56.9%)	254 (58.8%)	0.605
	Yes	116 (40.3%)	62 (43.1%)	178 (41.2%)	

There was no significant differences among three types of teeth in terms of frequency of gouging (Table 3). The prevalence of micro-leakage was the same in molar and anterior teeth but significantly higher in premolars. 

The frequency of various types of errors in teeth based on their location in the maxilla and mandible are presented in Table 4. The rightmost column of this test represents the level of significance of difference (based on *chi* square or Fisher’s exact test) for each error type in terms of the location of teeth in the maxilla and mandible. 

The prevalence of apical transportation was higher among the mandibular teeth than maxillary teeth. There was no significant differences between two jaws in apical perforation. 

Furthermore, 2.4% of the maxillary and 4.9% of the mandibular teeth suffered from gauging error. The difference between the two jaws in terms of gouging and ledge was not significant. Moreover, 1.4% of the maxillary teeth and 1.4% of the mandibular teeth had a ledge, and there was no significant differences between two jaws in terms of this variable. 

Finally, the assessment of improper temporary restoration using final radiography taken immediately at the end of treatment in the last visit, indicated that 40.3% of the maxillary teeth suffered from such a problem and 43.1% of the mandibular teeth had such a problem. Furthermore, the difference was not significant.

## Discussion

University clinic is the first for dentistry students to be trained and practice various skills required for a general dentist [12]. Therefore, it is of pivotal importance to assess the quality of education provided by the university by observing and investigating the treatments performed by the students [13]. The aim of the present study was to evaluate the endodontic treatments provided by fifth-year dentistry students of Hamadan universality of medical sciences in terms various error types, such as apical transportation, apical perforation, ledge formation, gauging, and improper temporary restoration. The quality of endodontic treatments was investigated by assessing the periapical radiographs taken before, during, and after such treatments had been performed [2, 14, 15].

Apical transportation was found in 19% of teeth that undergone an endodontic treatment, which is higher than those reported by other studies, such as Vukadinov *et al.* [7], Balto *et al.* [16], Haji-Hassani *et al.* [17], Smadi *et al.* [15]. In contrast, it was lower than those reported by some studies, including Alhekeir *et al.* [3], Moradi *et al.* [18], Eleftheriadis and Lambrianidis [4]. 

Probably the reason behind the lower rate of apical transportation in the study carried out by Vukadinov *et al.* [7] is that they utilized flexible nickel-titanium instruments and EDTA in curved canals. 

In the study carried out by Balto *et al.* [16], the same technique as used in the present study was employed, in which the step back method alongside a stainless steel file were utilized, but gates glidden sizes 2 to 4 were used for a direct access which can result in a lower rate of apical transportation [16]. Although the number of training hours provided by the Hamadan University of Medical Science was the same as the Jordan University (the study carried out by Smadi *et al.* [15], the occurrence of apical transportation was different between them, which can be due to the additional 28-h oral education implemented in the Jordan University, making dentistry students more familiar with the theoretical basis of endodontic treatments. 

In contrast to the present study, which used radiographs to investigate the endodontic errors, Alhekeir *et al.* [3] employed a self-reporting approach in this regard, so that they asked students to report their errors during endodontic treatment. Considering the limitation of two dimensional radiographs, the rate of errors in a self-reporting approach would be higher. Moradi *et al.* [18] used a passive step back approach; however, a higher level of error was reported by that study which can be due to the different definitions of apical transportation and ledge formation in these two studies, leading to different results. It is worth mentioning that Moradi *et al. *[18] regarded apical transportation and ledge formation as a single entity. 

Eleftheriadis and Lambrianidis [4] reported that the prevalence of apical transportation was significantly higher in teeth with severe curve, while in our university students are not permitted to treat such teeth, so there was no severely curved tooth among our samples. 

Regarding ledge formation, the rate reported by the present study was lower than those reported by other studies, including Smadi *et al.* [15], it means 5.2%, and Balto *et al.* [16], *i.e.* 14%, which can be due to the help provided by postgraduate students to modify such errors. 

In the Hamadan University of Medical Sciences dentistry students usually utilize the step back technique with stainless steel hand instruments for performing the endodontic treatments which is prone to apical transportation and ledge formation because they do not have good flexibility contrary to NiTi files [5]. To prevent such an error or reduce its probability, the use of NiTi files and step down and passive step back methods are proposed, in which the larger instruments are employed for canal orifice and then the smaller ones are utilized for reaching the apical region [3]. 

Apical perforation was observed in 17.6% of cases, much higher than those reported in other studies such as the one by Eleftheriadis and Lambrianidis [4], Yavari *et al.* [5], Smadi *et al.* [15], and Abraham SB [9]. In contrast, this rate of apical perforation occurrence was lower than those reported in some other studies, *e.g.* Alhekeir *et al. *[3], and comparable to what reported by Haji-Hassani *et al.* [17] and Mukhaimer [1]. The higher level apical perforation compared to that of Eleftheriadis and Lambrianidis [4] is reasonable because they explained in their study that cases with perforation had been referred to a specialized department, consequently they were dropped from the study. 

The difference between our results and what reported by Yavari *et al.* [5] is attributable to the teacher to student ratio (1 to 10 in our study *vs.* 1 to 5 reported by Yavari *et al.* [5]). The lower the ratio, the higher the supervision, and consequently the lower the opportunity for an error to be committed. 

The teacher/ratio reported in the study of Abraham SB [9] was more suitable than ours, also in the present study the postgraduate students and not professors were employed to supervise the performance of general dentistry students, so it can be inferred that they provide a better preclinical education for their students. Because of these, the prevalence of apical perforation was higher in our study. 

The difference between the prevalence of apical perforation between this study and Alhekeir *et al.* [3] is also due to, different methods for evaluating errors. Although in the present study errors were reported according to student’s reports not radiographs. 

The use of inflexible stainless steel instruments is another reason why the prevalence of apical perforation and apical transportation were high in the present study, while the use of nickel-titanium alloy for processing endodontic files because of its flexibility would result in a more favorable outcome, particularly for curved canals. Advantages of such an alloy have been illustrated by many studies [2, 4, 19]. Accordingly, the provision of the NiTi rotary technique for dentistry students is recommended, because the use of such instruments would lead to a better canal preparation [6]. It should be noted that using NiTi rotary instruments for undergraduate students has faced some resistance because of its costs and failure proneness [19]. 

The prevalence of apical transportation was higher in mandibular teeth than in maxillary teeth which is in line with previous studies [7, 11], may be because filing of mesial canals is harder. However, the prevalence of apical perforation and ledge formation were similar in mandibular and maxillary teeth. 

The prevalence of gauging was 3.2% with no difference between the anterior, premolar and molar teeth. This prevalence for this type of error is normal among dentistry students. The gauging is a result of too extended access cavity and an undermined enamel and dentin walls [7, 13]. The results are comparable with those reported by Balto *et al.* [16] and Haji-Hassani *et al.* [17]. Whereas, it was lower than some other studies [4, 11, 18]. Gharechahi *et al.* [18] explained that the difficulty of determining the calcified canal in molar teeth was the main reason for the high prevalence of the gouging error. However, it should be noted that in the Hamadan University of Medical Sciences, students are not permitted to work on teeth with calcification. 

According to the guidelines published by European Society of Endodontists, after completing endodontic treatment, in order to prevent bacterial contamination to be developed or tooth fracture, the teeth should be restored properly [13]. The quality of permanent coronal restoration has been studied by many researchers [20, 21]. However, because dentistry students normally performed a temporary restoration and not a permanent one, in the present study, we assessed the quality of this type of restoration performed by the fifth-year dentistry students. The gap between the gingival floor and temporary restoration in the radiograph was regarded as the improper temporary restoration. It was found that the prevalence improper restoration was unacceptably high and there is need for improving the education in this regard. 

According to previous studies conducted in this area, the skills of students in performing an endodontic treatment or root restoration depend on such factors as the time allocated to preclinical and clinical educations, teacher to student ratio, the competence of teachers, methods used for evaluating the students, and the number of students [5, 9, 22]. The significant effect of preclinical and clinical educations on the quality of endodontic treatment performed by students has been well documented [10, 23-25]. These studies have illustrated how the preclinical curriculum affects the ability of students in performing endodontic treatment in clinics. Accordingly, the modification of preclinical curriculum for reducing the occurrence rate various errors is recommended. Increasing the teacher to student ratio, increasing the time allocated to the preclinical education, the use of new instruments such as rotary machines, electronic apex locator, and implementing a new and systematic method for evaluating the theoretical and practical competence of students are recommended [9].

In retrospective studies some records will miss due to incomplete records, bad quality radiographs, so we recommend prospective studies in this field.

## Conclusion

The most prevalent type of error observed in teeth treated by fifth year dentistry students was the apical transportation. Molars were more prone to errors than other types of teeth. The quality of training provided for dentistry students should be improved and endodontic curriculum should be modified.
